# Associations between social factors and spinal pain-related disability in people with back pain and/or neck pain.

**DOI:** 10.1080/07853890.2025.2577877

**Published:** 2025-11-03

**Authors:** Lisette Bijker, Margreet ten Have, Gwendolyne G.M. Scholten-Peeters, Michel W. Coppieters, Pim Cuijpers, Leonore M. de Wit

**Affiliations:** a Department of Human Movement Sciences, Faculty of Behavioural and Movement Sciences, Vrije Universiteit Amsterdam, Amsterdam Movement Sciences - Program Musculoskeletal Health, The Netherlands; b Department of Clinical Psychology, Faculty of Behavioural and Movement Sciences, Vrije Universiteit Amsterdam, Amsterdam Public Health Research Institute, The Netherlands; c Trimbos Institute, Netherlands Institute of Mental Health and Addiction, Utrecht, The Netherlands; d School of Health Sciences and Social Work, Griffith University, Brisbane, Australia

**Keywords:** Musculoskeletal pain, rehabilitation, psychosocial factors, pain-related disability, population study

## Abstract

**Purpose:**

Pain-related disability is often managed using the biopsychosocial model. However, social factors, such as living situation, financial hardship and employment status, are understudied. This study focused on the association between social factors and pain-related disability after correcting for important biological factors (e.g. BMI) and psychological factors (e.g. depression) in people with spinal pain.

**Methods:**

Cross-sectional data were used from the fourth wave (*N* = 4,007) of the second Netherlands Mental Health Survey and Incidence Study (NEMESIS-2), a representative cohort study in the general population of The Netherlands. Data from adults between 27–73 years of age with spinal pain (*N* = 1,451) were extracted. Data were analysed using multivariable linear regression analyses and results are expressed in (standardized) beta coefficients.

**Results:**

Loneliness (β = 0.06; *p* = 0.01) and financial hardship (β = 0.04; *p* = 0.04) were significantly associated with pain-related disability after correction for biological and psychological factors. Educational background and employment status were the significant effect modifiers for the association of pain intensity and pain-related disability.

**Discussion:**

The social dimension acts as an independent component influencing pain-related disability, establishing itself as an important domain within the biopsychosocial model meriting deeper exploration. Clinicians and researchers should explore these social factors more in people with spinal pain in management and research.

## Introduction

Low back pain and neck pain, that is spinal pain, are both in the top-ten of most common somatic disorders worldwide [1,2]. Spinal pain contributes significantly to disability, resulting in substantial societal costs [3]. International guidelines emphasize the importance of working within a biopsychosocial paradigm to consider the intricate interplay of factors in the onset and maintenance of spinal pain [4–6].

Previous research showed that factors in the biological domain are associated with pain-related disability, such as higher pain intensity, higher body-mass index and low levels of physical activity [7]. Factors in the psychological domain are associated with pain-related disability as well, such as higher levels of depression, pain catastrophising and fear-avoidance beliefs [7]. In contrast to these two domains, the social domain has hardly been studied in spinal pain [8]. Nevertheless, an increasing number of researchers and clinicians argue that social factors play a fundamental role in pain-related disability [9,10].

Social factors impacting disability can be categorized in five domains: (1) gender, (2) socioeconomic position, (3) social relationships, (4) residential and community context, and (5) race, ethnicity, and cultural context [11]. For any type of disability, increases in the number of social risk factors are associated with higher odds of disability [12] and this pattern occurs in people with specific pain conditions such as arthritis [13] and following lumbar spine revision surgery [14]. Most studies on social factors associated with spinal pain focused on socio-economic position or social relationships [15–17]. However, these studies typically examined the association between social factors and the presence of spinal pain rather than their association with pain-related disability. They demonstrate that people who are separated or widowed, or who have a low income or a low level of education are more likely to experience spinal pain (15, 16, 17). Financial concerns, a frequently overlooked social factor and a significant source of daily stress, have been associated with spinal pain [18]. Even fewer studies have evaluated social isolation and loneliness quantitatively, even though in qualitative studies people with spinal pain experiencing loneliness or social isolation indicate that these factors play a substantial role in their experience of pain [19,20,21]. The evidence for the role of social support in spinal pain is inconclusive according to a systematic review [22]. It seems that there is moderate evidence that low levels of informal social support are related to psychological health outcomes such as depression, kinesiophobia and pain catastrophising. However, these are related to the prevalence of spinal pain but not spinal pain-related disability directly. Providing informal care for another person is another factor, which is associated with psychological health, such as depression [23], although this is not yet investigated for spinal pain [24].

Furthermore, most studies that assessed the relationship between a social factor and spinal pain did not correct for biological and psychological factors (15, 18, 25]. Correcting for other confounding factors from the biological domain (such as pain intensity and lifestyle-related factors) and the psychological domain (such as; depression and anxiety) seems prudent as these factors might influence the association between social factors and pain-related disability, as was the case for social support [22].

This study aimed to assess the association between social factors and pain-related disability among people with spinal pain (back pain and/or neck pain) in the general population, while correcting for biological and psychological factors. Improved knowledge about the association between social factors and pain-related disability may provide clarity for clinicians and researchers which social factors should be considered in spinal pain management [26].

## Methods

### Study design

This study used a cross-sectional design and was reported in accordance with the STROBE-checklist (Strengthening the Reporting of Observational Studies in Epidemiology; [27]). Data were extracted from The Netherlands Mental Health Survey and Incidence Study − 2 (NEMESIS-2), a large population-based cohort study in The Netherlands [28]. NEMESIS-2 commenced in 2007 based on a multistage, stratified, random sampling procedure [28]. An extensive description of the design, sampling procedures, interview methods and instruments has been provided elsewhere (de Graaf et al. 2010). All participants signed written informed consent prior to participation. NEMESIS-2 was approved by the Medical Ethics Review Committee for Institutions on Mental Health Care (METIGG) in 2007 in Tilburg, The Netherlands (NL18210.097.07) and adheres to the principles stated in the ‘Declaration of Helsinki’ [29].

### Participants

Potential participants were included in NEMESIS-2 if they were between 18 and 64 years of age at baseline, and proficient in Dutch. In the first wave (T0), conducted from November 2007 to July 2009, 6,646 individuals were interviewed (response rate 65.1%; average duration interview: 95 min). The sample was broadly nationally representative, although younger participants were somewhat underrepresented (De Graaf et al. 2010). All respondents were approached for follow-up after 3 years (T1: *n* = 5,303; response rate 80.4%, with those deceased excluded; interview duration: 84 min), after 6 years (T2: *n* = 4,618; response rate compared to T1: 87.8%; interview duration: 83 min) and after 9 years (T3: *n* = 4,007; response rate compared to T2: 87.7%; interview duration: 101 min). Attrition between T0 and T3 was not significantly associated with any of the assessed 12-month mental disorders at T0 after controlling for sociodemographic characteristics (De Graaf et al. 2018). For this study, data from T3 were extracted as there was no information regarding pain-related disability and pain site in the first waves. At T3, 1,451 participants (36.2%) had low back pain or neck pain and reported either one of these pain sites as their primary pain site in the six months prior to the moment of interview and were therefore included for this study.

### Pain-related disability

The primary outcome of this study was pain-related disability, which was measured with subscale 2 of the Graded Chronic Pain Scale (GCPS) [30] at T3. This subscale consists of three questions regarding experienced disability due to pain in the last six months in (1) daily activities, (2) recreational, social and family activities and (3) work (including housework) activities, each scored on an 11-point Likert scale [0 = no limitations, 10 = severely limited]. It is calculated by the total of three questions, divided by the number of questions, and multiplied by 10 [range: 0–100]. The second subscale of the GCPS is a reliable instrument to assess pain-related disability in the general population [31] (Hawker et al. 24).

### Social factors

Included social factors were mostly assessed with one question. These include living situation (with a partner; no partner), educational level (primary, basic vocational or lower secondary; higher secondary or lower professional education; higher professional or university), financial hardship (‘Do you have adequate financial resources to cover your expenses for the month’ Yes; no), being an informal caregiver (‘Have you provided non-professional care for a family member, partner, friend or acquaintance who needed assistance due to a mental or physical disability, an intellectual disability or old age in the last 12 months?’ yes; no) and employment status (unemployed or unable to work; employed; retired). Additionally, loneliness was measured with the Loneliness Scale [32]. This scale consists of 11 items (range 0–11, higher scores indicate higher levels of loneliness) and in the general population its internal consistency is high (alpha = 0.88) [33].

### Demographical, biological, and psychological factors

Potentially relevant confounders were selected based on previous research (25, 34,35,36,37,38,39] and consensus on inclusion of the confounders was reached following a focus meeting of experts in the field of psychology (LB, LW, PC), musculoskeletal pain disorders (GGMSP, MWC) and epidemiology (MH). The included potential confounders are described in Table 1.

**Table 1.** Potential confounders.

	Potential confounders	Measurement
**Demographics**	Age	In years
	Sex	Male / female
**Biological factors**	Pain intensity	Pain intensity was measured with subscale 1 of the Chronic Pain Graded Scale [30,40]. This subscale consists of three questions (current pain intensity, pain intensity of most severe episode in the past six months, average pain intensity past six months) scored on an 11-point Likert Scale [0 = no pain, 10= extreme pain]. It is calculated by the total of each question divided by the number of questions and multiplied by 10 [range: 0–100]. The CPGS is reliable [31]. The internal consistency within the NEMESIS-2 study is considered good (Cronbach’s alpha = 0.75; de Graaf et al, 2010).
	BMI	Calculated with participant’s (self-reported) height (cm) and weight (kg). BMI = kg/m^2^
	Somatic comorbid disorder	A list of 16 common chronic somatic disorders was presented to the participants (including asthma, COPD, vascular problems, diabetes, rheumatic, arthritis, migraine, visual or audiotory impairments, etc.). The response was dichotomised as present if the participant was treated or monitored by a medical doctor in the past 12 months or absent.
**Psychological factors**	Depressive symptoms	Depressive symptoms in the past week were assessed using the ‘Center for Epidemiologic Studies - Depression – Short Form’ (CES-D-short; 41]. This questionnaire consisted of 10 items using a four-point Likert Scale [0–3] and is reliable [42]. The internal consistency within the NEMESIS-2 study is considered good (Cronbach’s alpha = 0.77; de Graaf et al, 2010).
	Anxiety symptoms	Anxiety symptoms in the past 4 weeks were assessed using the Anxiety subscale of the ‘Hospital Anxiety and Depression Scale’ (HADS-A; 43]. This seven-item subscale is scored on a 4-point Likert scale [0–3] and is reliable [44]. The internal consistency within the NEMESIS-2 study is considered good (Cronbach’s alpha = 0.76; de Graaf et al., 2010)
	Insomnia	Insomnia in the past 4 weeks was assessed with the ‘Women’s Health Initiative Insomnia Rating Scale’ (WHI-IRS). The scale consists of five items using a five-point Likert Scale [1–5] and is reliable [45]. The internal consistency within the NEMESIS-2 study is considered good (Cronbach’s alpha = 0.76; de Graaf et al., 2010).
**Lifestyle-related factors**	Physical activity	Measured with one question based on the ‘International Physical Activity Questionnaire (IPAQ; 46]; ‘Think about an average week in the last 12 months and add up the minutes that you were physically active on average (during or outside of work). How many days were you physically active for at least 30 minutes per day on average?’ Physical activities were further clarified as activities that would result in an elevated heart rate, such as exercise, a brisk walk, cycling or rigorous cleaning. This response was coded dichotomous as physically active according to the ‘Dutch norm of Physical Activity’ (5 or more days per week) or physically inactive (less than 5 days per week).
	Alcohol use	Alcohol use was measured as the number of alcoholic drinks per week on average in the past 12 months.

### Statistical analyses

Descriptive statistics are presented as proportions, means and standard deviations. Assumptions for performing multivariable linear regression were checked (linear relationship between dependent and independent variables, normal distribution of the residuals and multicollinearity (VIF > 10)). First, the crude main effects for the association between individual social factors and pain-related disability were calculated. Following the univariable analyses, two models were built: Model 1 explored the association between the social factors and pain-related disability corrected for age and sex. In Model 2, we additionally corrected for biological factors (BMI, physical activity, somatic comorbid disorders and pain intensity) and psychological factors (depressive symptoms, anxiety symptoms, insomnia and alcohol use). Lastly, the effect modification was assessed to explore whether the associations of sex (male vs. female) [47], pain site (neck vs. back vs. neck and back) [48] and pain intensity (high vs. low) [10] with pain-related disability differed across levels of social factors. The associations were expressed as the beta-coefficient (B), with 95% confidence intervals, standard errors, *p*-values and adjusted-beta0coefficient (ß). The association expressed by ß can be considered low (<0.3), moderate (>0.3 and <0.5) or high (>0.5) [49]. For all statistical tests, an alpha less than 0.05 was considered significant. All statistical analyses were performed using STATA version 16.1 (StataCorp, College Station, Texas, USA).

## Results

### Characteristics of the sample

The prevalence of spinal pain as the primary pain location in the 4th wave (T3) of the NEMESIS-2 study was 36.2% (*N* = 1,451 from *N* = 4007). The participants’ mean (SD) pain-related disability (0–100) score was 25.53 (SD = 24.38), mean (SD) pain intensity (0–100) was 44.36 (SD = 17.52) and they experienced an average of 76.25 (SD = 72.8) days living with pain within the past six months (180 days). A total of 29.9% of participants scored above the cut-off for having the clinically elevated symptoms of insomnia (≥8), 9.3% reported having clinically elevated symptoms of depression (≥10) and 4.6% reported having clinically elevated anxiety symptoms (≥8). Almost 75% lived together with a partner and 65% had a paid job. An overview of the characteristics of this sample is presented in Table 2.

**Table 2.** Characteristics of the sample (*N* = 1,451).

	Variable [range]	Mean (SD)
**Demographics**	Age in years	53.68 (12.19)
	Sex (Female, %)	57.55
**Biological factors**	Pain-related disability [0–100]	25.53 (24.38)
	Pain intensity [0–100]	44.36 (17.52)
	Number of days with pain in the past 6 months [0–180]	76.25 (1.91)
	Pain medication (%) Every day Every week Couple of times a month A couple of times in the past six months None	11.30 7.79 10.27 18.06 52.52
	Pain location (%) Back pain only Neck pain only Back and neck pain	61.61 21.02 17.37
	BMI [16.94–49.54]	26.13 (4.57)
	Somatic comorbid disorder (Yes, %)	40.45
**Psychological factors**	Depressive symptoms – CES-D-short [0–30, cut-off ≥10]	4.30 (3.81)
	Anxiety symptoms – HADS-A [0–24, cut-off ≥8]	2.40 (2.29)
	Insomnia – WHI-IRS [5–25, cut-off ≥8]	6.48 (4.57)
**Lifestyle-related factors**	Alcohol use (%) No Mild Moderate Excessive Physically active (Yes, %)	22.81 55.69 16.47 5.03 38.29
**Social factors**	Living situation (with partner, %)	73.88
	Educational Level (%) Primary / Lower secondary/ basic vocational Higher secondary Higher professional / university	28.95 30.39 40.66
	Employment status (%) Employed Homemaker Unemployed/ unable to work Retired, other	62.70 7.34 9.90 20.07
	Financial hardship (Being unable to make ends meet, %)	4.15
	Loneliness - LS [0–11]	1.54 (2.29)
	Being an informal caregiver (Yes, %)	38.59

SE = standard error; CES-D-short = Center for Epidemiologic Studies- Depression – Short Form; HADS-A = Hospital Anxiety and Depression Scale-Anxiety scale; WHI-IRS = Women’s Health Initiative Insomnia Rating Scale; LS = Loneliness Scale.

### Associations between social factors and spinal pain-related disability

All of the assumptions for performing multivariable linear regression were checked and met prior to the analysis: there was a linear relationship between dependent and independent variables, a normal distribution of the residuals of the dependent variable and no evidence of multicollinearity (VIF > 10)). Table 3 shows the association between social factors and pain-related disability for both models (Model 1 and Model 2) expressed as beta-coefficients, confidence interval, standard error, standardized beta and statistical significance. In Model 1 (corrected for age and sex), all social factors were significantly associated with pain-related disability apart from being a non-professional caregiver (Table 3). In Model 2 (corrected for age, sex, biological and psychological confounders), a number of social factors remained significantly associated with pain-related disability, namely loneliness, employment situation, educational background and financial hardship.

**Table 3.** Association between social factors and pain disability.

		Model 1				Model 2			
Variable	Category	B (CI)	SE	ß	*P* value	B (CI)	SE	ß	*P* value
Loneliness		2.09 (1.59–2.59)	0.26	0.21	<0.001	0.62 (0.14–1.11)	0.25	0.06	0.01
Educational level	Primary education	REF	REF	REF	REF	REF	REF	REF	REF
	Higher secondary education	−4.71 (−7.96 - −1.46)	1.66	−0.09	0.005	−2.74 (−5.39- −0.09)	1.35	−0.05	0.043
	Higher professional education; university	−10.27 (−13.34- −7.21)	1.56	−0.21	<0.001	−3.36 (−5.92- −0.80)	1.30	−0.07	0.010
Living situation	No partner	2.87 (0.05–5.69)	1.44	0.05	0.046	0.52 (−1.79- 2.83)	1.18	0.009	0.662
Employment situation	Working	REF	REF	REF	REF	REF	REF	REF	REF
	Homemaker	4.18 (−0.81-9.17)	2.54	0.04	0.10	−0.06 (−4.15 − 4.04)	2.09	−0.0006	0.98
	Unemployed/disabled	18.63 (14.40– 22.85)	2.16	0.23	<0.001	5.85 (2.21– 9.48)	1.85	0.07	0.002
	Retired / other	3.27 (−0.86 − 7.39)	2.10	0.05	0.12	−0.35 (−3.76 − 3.05)	1.74	−0.006	0.84
Being an informal caregiver		−1.15 (−3.74 − 1.44)	1.32	−0.02	0.39	−1.17 (−3.25 − 0.91)	1.06	−0.02	0.27
Financial hardship	Adequate financial recourses to cover the month? (Yes)	16.03 (9.88– 22.18)	3.13	0.13	<0.001	5.30 (0.22 –10.38)	2.59	0.04	0.04

B = Beta-coefficient; ß = standardized beta-coefficient; CI = confidence interval; SE = standard error, REF = reference category.

Model 1= corrected for age and sex; model 2 = corrected for age, sex, biological- and psychological confounders.

All statistically significant social factors of pain-related disability (in Model 2) were analysed for the potential effect modification of the association between sex, pain location and pain intensity, and pain-related disability. The associations between sex or pain location and pain-related disability were not dependent on social factors. Education (higher professional education vs. primary education only: *ß = 0.17, p = 0.01*) and employment situation (being unemployed/unable to work versus employed: *B = 0.21, SE = 0.09, ß = 0.15, p = 0.025*; being retired versus employed: *B = 0.26, SE = 0.07, ß = 0.22, p < 0.001*) modified the association between pain intensity and pain-related disability.

In Table 4, the association between pain intensity and spinal pain-related disability is expressed per subgroup in beta-coefficient, confidence interval, standard error, standardized beta and statistical significance. For a high educational background, the association between pain intensity and pain-related disability is less strong compared to people with a primary education only. For employment situation, the association between pain intensity and pain-related disability is stronger for people who are unemployed, disabled or retired compared to employed people.

**Table 4.** Association between pain intensity and pain disability.

Social factor	Group	B (CI)	SE	ß	*P*-value
Educational level	Primary education	0.79 (0.67–0.91)	0.06	0.55	<0.01
	Higher secondary education	0.75 (0.63–0.87)	0.06	0.51	<0.01
	Higher professional education; university	0.59 (0.50–0.68)	0.05	0.46	<0.01
Employment situation	Working	0.61 (0.53–0.69)	0.04	0.45	<0.01
	Homemaker	0.70 (0.43–0.98)	0.14	0.46	<0.01
	Unemployed/unable to work	0.74 (0.55–0.95)	0.10	0.53	<0.01
	Retired / other	0.85 (0.73–0.98)	0.06	0.62	<0.01

B = Beta-coefficient; ß = standardized beta-coefficient; CI = confidence interval; SE = standard error.

## Discussion

The aim of this study was to assess the association between social factors (loneliness, educational background, non-professional caregiving, living arrangement, employment and financial situation) and pain-related disability in people with back pain and/or neck pain from the general population, while correcting for important biological and psychological factors. Our results showed that higher levels of loneliness, lower level of education, being retired or unemployed/unable to work and experiencing financial hardship were significantly associated with pain-related disability when corrected for biological and psychological factors, although the associations were small. These associations did not differ between sex, nor between back pain and/or neck pain. Employment status and educational level were found to be statistically significant effect modifiers for pain intensity in relation to pain-related disability resulting in moderate-to-high associations.

These findings are in line with several cross-sectional studies examining associations of social factors with the prevalence of spinal pain, though not specifically with spinal pain-related disability [15–17]. In particular, a previous study performed in the US among 5,103 adults (of which 700 people experienced persistent low back pain) found that unemployment and a low educational level modified the association between pain intensity with persistent low back pain as well [17]. Although this study did not correct for psychological confounders, they found the associations between behavioural, psychological and medical issues and persistent low back pain in their sample as well. Another study with over 33.000 adults, of which approximately one-third experienced persistent spinal pain in the US (18) found financial worries were associated with the occurrence of persistent spinal pain, even when controlled for socio-economic status. However, this study did not control for psychological factors either. Similar findings were observed in a study in Brazil [15] with 600 adults of which 20% experienced neck pain. This study also showed that being separated or widowed, having a low income and low educational level are associated with neck pain. A study that did correct for health status and psychological factors used pain interference as their dependent variable [16]. This study also showed the association between socioeconomic status and pain interference, but did not specify a pain location.

The finding that is not in line with other cross-sectionals studies is the prevalence of spinal pain in this cohort which is quite high at 36%. This is higher than the 1-month prevalence of 23% found in a global systematic review of low back pain [50 or 27% for neck pain [51]. Although this sample is nationally representative of the population in the Netherlands [52], the third wave of this study excludes participants between 18 and 26 years of age due to the follow-up period. Also, this study included people with both low back pain and neck pain. As the prevalence of spinal pain increases with age, this might explain the high prevalence found in this sample [50].

An important finding of this study is that it demonstrates that social factors are independent contributors to pain-related disability among adults with spinal pain and that their effects are not fully accounted for by biological or psychological factors. It appears that within the domains of musculoskeletal health and pain, social factors do not receive adequate acknowledgment when combined with the psychological factors and collectively referred to as psychosocial factors. Secondly, this study assessed the relationship between social factors and pain-related disability, as opposed to the prevalence of (persistent) spinal pain that is more commonly used in cross-sectional studies [15,16,17]. Spinal pain-related disability is the recommended primary outcome in core outcome sets for spinal pain [53] as it encapsulates a quantitative scale for physical functioning in daily life.

There are also some limitations in this study. Firstly, the cross-sectional design of the study prevents the ability to establish causality. Secondly, we lacked information about the duration of neck pain or back pain and the number of episodes, and consequently, we could not distinguish different associations between people with acute, recurrent or persistent pain. We also lacked data regarding type, dose and intended use of pain medication which is why it was not included as a covariate. Both of these limitations are due to the existing dataset being more focussed on mental health in the general population rather than on spinal pain. Thirdly, a limitation of this study is the use of data collection tools with varying recall periods, which may introduce recall bias and affect the accuracy of the results. Fourth, although this study corrected for biological factors and psychological factors, there might be other factors that are important confounders that were not included in the existing dataset, such as health literacy [54] and work-related factors. With regards to health literacy, it might be that people with a higher educational level are more able to find, understand and use information and services to inform health-related decisions for themselves, thereby profiting more from the treatment of spinal pain. Another important category of confounders worth further exploration that were not included in this study is work-related factors, such as job demands, physically demanding jobs, support in the workplace, job autonomy and job satisfaction [55,56,57]. Although they would only be applicable to the working population, these factors might still influence the strength of the association between the social factors in this study and spinal pain-related disability. Finally, social risk factors affecting health manifest in various forms. The identify five domains: gender, socioeconomic position, social relationships, residential and community context, and race, ethnicity, and cultural context [11]. Factors associated with the first three domains were included in this study.

Although our study is explorative in nature, it contributes to the emerging awareness of the social domain as an important domain in the biopsychosocial model. Traditionally, there has been more emphasis on the biological factors associated with spinal pain [7]. As our understanding of pain as a global health problem continues to evolve, the role of psychological and social factors in spinal pain-related disability is becoming more evident and confirmed in more recent publications [7]. It appears that the time is opportune to integrate social factors, such as loneliness and financial situation, as well as socio-economic factors, such as education and work-related factors, into research and clinical practice. For healthcare practitioners, it seems prudent to become more aware of these factors in the management of patients when assessing and managing spinal pain.

## Data Availability

Data is available upon reasonable request at the Trimbos Institute in Utrecht, the Netherlands by contacting the corresponding author.

## References

[1] Sebbag E, Felten R, Sagez F, et al. The world-wide burden of musculoskeletal diseases: a systematic analysis of the World Health Organization Burden of Diseases Database. Ann Rheum Dis. 2019;78(6):844–848. doi:10.1136/annrheumdis-2019-215142. 30987966

[2] GBD 2021 Neck Pain Collaborators. Global, regional, and national burden of neck pain, 1990-2020, and projections to 2050: a systematic analysis of the Global Burden of Disease Study 2021. Lancet Rheumatol. 2024;6(3):e142–e155. doi:10.1016/S2665-9913(23)00321-1. 38383088 PMC10897950

[3] Park PW, Dryer RD, Hegeman-Dingle R, et al. Cost Burden of chronic pain patients in a large integrated delivery system in the United States. Pain Pract. 2016;16(8):1001–1011. doi:10.1111/papr.12357. 26443292

[4] Corp N, Mansell G, Stynes S, et al. Evidence-based treatment recommendations for neck and low back pain across Europe: a systematic review of guidelines. Eur J Pain. 2021;25(2):275–295. doi:10.1002/ejp.1679. 33064878 PMC7839780

[5] Leach MJ, Climstein M, Fryer G, et al. Mapping guideline-informed care for chronic non-specific low back pain with the biopsychosocial approach: a rapid review. Pain Pract. 2023;23(5):543–552. doi:10.1111/papr.13214. 36853009

[6] Oliveira CB, Maher CG, Pinto RZ, et al. Clinical practice guidelines for the management of non-specific low back pain in primary care: an updated overview. Eur Spine J. 2018;27(11):2791–2803. doi:10.1007/s00586-018-5673-2. 29971708

[7] Hartvigsen J, Hancock MJ, Kongsted A, et al. What low back pain is and why we need to pay attention. Lancet. 2018;391(10137):2356–2367. doi:10.1016/S0140-6736(18)30480-X. 29573870

[8] Mescouto K, Olson RE, Hodges PW, et al. A critical review of the biopsychosocial model of low back pain care: time for a new approach? Disabil Rehabil. 2022;44(13):3270–3284. doi:10.1080/09638288.2020.1851783. 33284644

[9] Craig KD, MacKenzie NE. What is pain: are cognitive and social features core components? Paediatr Neonatal Pain. 2021;3(3):106–118. doi:10.1002/pne2.12046. 35547951 PMC8975232

[10] Mugoya GC, Hooper LM, Tomek S, et al. The interrelationships among pain interference, depressive symptoms, loneliness, and employment status: a moderated mediation study. Clin Rehabil. 2018;32(7):967–979. doi:10.1177/0269215518758483. 29457478

[11] National Academies of Sciences, Engineering, and Medicine. Accounting for social risk factors in medicare payment. Washington, DC: the National Academies Press; 2017. doi:10.17226/23635. 28617569

[12] Sharpe JA, Miller R, Cook CE, et al. Social risk factors are associated with disability prevalence - results from 17 states in the 2017 behavioral risk factor surveillance system. Am J Health Promot. 2023;37(4):453–463. doi:10.1177/08901171221132390. 36194861 PMC11862991

[13] Rethorn ZD, Rethorn TJ, Cook CE, et al. Association of burden and prevalence of arthritis with disparities in social risk factors, findings from 17 US states. Prev Chronic Dis. 2022;19: E08. doi:10.5888/pcd19.210277. 35175917 PMC8880108

[14] Buck E, Rethorn ZD, Garcia AN, et al. The collective influence of social determinants of health on individuals who underwent lumbar spine revision surgeries: a retrospective cohort study. World Neurosurg. 2022;165:e619–e627. Epub 2022 Jun 27. PMID: 35772707. doi:10.1016/j.wneu.2022.06.107. 35772707

[15] Genebra CVDS, Maciel NM, Bento TPF, et al. Prevalence and factors associated with neck pain: a population-based study. Braz J Phys Ther. 2017;21(4):274–280. doi:10.1016/j.bjpt.2017.05.005. 28602744 PMC5537482

[16] Lacey RJ, Belcher J, Croft PR. Does life course socio-economic position influence chronic disabling pain in older adults? A general population study. Eur J Public Health. 2013;23(4):534–540. doi:10.1093/eurpub/cks056. 22874735 PMC3719471

[17] Shmagel A, Foley R, Ibrahim H. Epidemiology of chronic low back pain in US adults: national Health and Nutrition Examination Survey 2009–2010. Arthritis Care Res. 2016;68(11):1688–1694. doi:10.1002/acr.22890. PMC5027174 26991822

[18] Yang H, Haldeman S. Chronic spinal pain and financial worries in the US adult population. Spine (Phila Pa 1976). 2020;45(8):528–533. doi:10.1097/BRS.0000000000003319. 31770336

[19] Froud R, Patterson S, Eldridge S, et al. A systematic review and meta-synthesis of the impact of low back pain on people’s lives. BMC Musculoskelet Disord. 2014;15(1):50. doi:10.1186/1471-2474-15-50. 24559519 PMC3932512

[20] Kennedy H, Harvie DS, Coppieters MW. Do threats and reassurances reside in the biological, psychological or social domain? A qualitative study in adults and young people with chronic pain. Br J Pain. 2024;18(6):472–481. doi:10.1177/20494637241263291. 39552922 PMC11561933

[21] MacNeela P, Doyle C, O’Gorman D, et al. Experiences of chronic low back pain: a meta-ethnography of qualitative research. Health Psychol Rev. 2015;9(1):63–82. doi:10.1080/17437199.2013.840951. 25793491

[22] Campbell P, Wynne-Jones G, Dunn KM. The influence of informal social support on risk and prognosis in spinal pain: a systematic review. Eur J Pain. 2011;15(5):444.e1-14–444.14. doi:10.1016/j.ejpain.2010.09.011. PMC3142815 20970363

[23] Torres Á, Blanco V, Vázquez FL, et al. Prevalence of major depressive episodes in non-professional caregivers. Psychiatry Res. 2015;226(1):333–339. doi:10.1016/j.psychres.2014.12.066. 25667119

[24] Gomes NP, Pedreira LC, Gomes NP, et al. Health-related consequences of caring for dependent relatives in older adult caregivers. Rev Esc Enferm USP. 2019;53:e03446. Erratum in: rev Esc Enferm USP. 2019 Dec 02;53:e03536. doi:10.1590/S1980-220X2018002303446. 30864620

[25] Kirsch Micheletti J, Bláfoss R, Sundstrup E, et al. Association between lifestyle and musculoskeletal pain: cross-sectional study among 10,000 adults from the general working population. BMC Musculoskelet Disord. 2019;20(1):609. doi:10.1186/s12891-019-3002-5. 31847824 PMC6918691

[26] Bernstetter A, Brown NH, Fredhoff B, et al. Reporting and incorporation of social risks in low back pain and exercise studies: a scoping review. Musculoskelet Sci Pract. 2025;. 2025;77:103310. doi:10.1016/j.msksp.2025.103310. 40127512

[27] Von Elm E, Altman DG, Egger M, et al. The strengthening the reporting of observational studies in epidemiology (STROBE) statement: guidelines for reporting observational studies. J Clin Epidemiol. 2008;61(4):344–349. doi: 10.1016/j.jclinepi.2007.11.008 18313558 18313558

[28] World Medical Association. World Medical Association Declaration of Helsinki: ethical principles for medical research involving human subjects. JAMA. 2013;310(20):2191–2194. doi:10.1001/jama.2013.281053. 24141714

[29] De Graaf RON, Ten Have M, Van Dorsselaer S. The Netherlands mental health survey and incidence study-2 (NEMESIS-2): design and methods. Int J Methods Psychiatr Res. 2010;19(3):125–141. doi: 10.1002/mpr.317 20641046. 20641046 PMC6878518

[30] von Korff M, Ormel J, Keefe FJ, et al. Grading the severity of chronic pain. Pain. 1992;50:133–149. 1408309 10.1016/0304-3959(92)90154-4

[31] von Korff M. (2001) Epidemiologic and surver methods: chronic pain assessment in handbook of pain assessment. 2nd Edition.New York: Guilford Press.

[32] De Jong Gierveld J, Van Tilburg T. Manual of the loneliness scale. Amsterdam: Vrije Universiteit Amsterdam; 1999.

[33] de Jong Gierveld J, van Tilburg T. The De Jong Gierveld short scales for emotional and social loneliness: tested on data from 7 countries in the UN generations and gender surveys. Eur J Ageing. 2010;7:121–130. doi:10.1007/s10433-010-0144-6. 20730083 PMC2921057

[34] Ferreira PH, Beckenkamp P, Maher CG, et al. Nature or nurture in low back pain? Results of a systematic review of studies based on twin samples. Eur J Pain. 2013;17(7):957–971. doi:10.1002/j.1532-2149.2012.00277.x. 23335362

[35] Van Looveren E, Bilterys T, Munneke W, et al. The Association between sleep and chronic spinal pain: a systematic review from the last decade. J Clin Med. 2021;10(17):3836. doi:10.3390/jcm10173836. 34501283 PMC8432009

[36] Shiri R, Karppinen J, Leino-Arjas P, et al. The association between obesity and low back pain: a meta-analysis. Am J Epidemiol. 2010;171(2):135–154. doi:10.1093/aje/kwp356. 20007994

[37] Taylor JB, Goode AP, George SZ, et al. Incidence and risk factors for first-time incident low back pain: a systematic review and meta-analysis. Spine J. 2014;14(10):2299–2319. doi:10.1016/j.spinee.2014.01.026. 24462537

[38] Tseli E, Boersma K, Stålnacke B-M, et al. Prognostic factors for physical functioning after multidisciplinary rehabilitation in patients with chronic musculoskeletal pain: a systematic review and meta-analysis. Clin J Pain. 2019;35(2):148–173. doi:10.1097/AJP.0000000000000669. 30371517 PMC6343958

[39] Zhang TT, Liu Z, Liu YL, et al. Obesity as a risk factor for low back pain: a meta-analysis. Clin Spine Surg. 2018;31(1):22–27. doi:10.1097/BSD.0000000000000468. 27875413

[40] Hawker GA, Mian S, Kendzerska T, Measures of adult pain: visual Analog Scale for Pain (VAS Pain), Numeric Rating Scale for Pain (NRS Pain), McGill Pain Questionnaire (MPQ), et al. Short-Form McGill Pain Questionnaire (SF-MPQ), Chronic Pain Grade Scale (CPGS), Short Form-36 Bodily Pain Scale (SF-36 BPS), and Measure of Intermittent and Constant Osteoarthritis Pain (ICOAP). Arthritis Care Res (Hoboken). 2011;63 Suppl 11(Suppl 11):S240–S52. doi:10.1002/acr.20543. 22588748

[41] Balsamo M, Cataldi F, Carlucci L, et al. Assessment of late-life depression via self-report measures: a review. Clin Interv Aging. 2018;10. 10.2147/CIA.S178943 PMC6199213 30410319

[42] Björgvinsson T, Kertz SJ, Bigda-Peyton JS, et al. Psychometric properties of the CES-D-10 in a psychiatric sample. Assessment. 2013;20(4):429–436. doi:10.1177/1073191113481998. 23513010

[43] Zigmond AS, Snaith RP. The hospital anxiety and depression scale. Acta Psychiatr Scand. 1983;67(6):361–370. doi:10.1111/j.1600-0447.1983.tb09716.x. 6880820

[44] Spinhoven P, Ormel J, Sloekers PP, et al. A validation study of the Hospital Anxiety and Depression Scale (HADS) in different groups of Dutch subjects. Psychol Med. 1997;27(2):363–370. PMID: 9089829. doi:10.1017/s0033291796004382. 9089829

[45] Levine DW, Kripke DF, Kaplan RM, et al. Reliability and validity of the women’s health initiative insomnia rating scale. Psychol Assess. 2003;15(2):137–148. doi:10.1037/1040-3590.15.2.137. 12847774

[46] Craig CL, Marshall AL, Sjöström M, et al. International physical activity questionnaire: 12-country reliability and validity. Med Sci Sports Exerc. 2003;35(8):1381–1395. doi:10.1249/01.MSS.0000078924.61453.FB. 12900694

[47] Coggon D, Ntani G, Walker-Bone K, et al. Epidemiological differences between localized and nonlocalized low back pain. Spine (Phila Pa 1976). 2017;42(10):740–747. doi:10.1097/BRS.0000000000001956. 27820794 PMC5418102

[48] Artus M, Campbell P, Mallen CD, et al. Generic prognostic factors for musculoskeletal pain in primary care: a systematic review. BMJ Open. 2017;7(1):e012901. doi: 10.1136/bmjopen-2016-012901 28096253 PMC5253570 28096253

[49] Cohen J. Statistical power analysis for the behavioral sciences. 2nd ed. Hillsdale, NJ: Lawrence Erlbaum Associates; 1988.

[50] HoyD, Bain C, Williams G, et al. A systematic review of the global prevalence of low back pain. Arthritis Rheum. 2012;64(6):2028–2037. doi: 10.1002/art.3434722231424. 22231424

[51] Kazeminasab S, Nejadghaderi SA, Amiri P, et al. Neck pain: global epidemiology, trends and risk factors. BMC Musculoskelet Disord. 2022;23(1):26. doi:10.1186/s12891-021-04957-4. 34980079 PMC8725362

[52] De Graaf RON, Ten Have M, Van Gool C, et al. Prevalence of mental disorders and trends from 1996 to 2009. Results from the Netherlands mental health survey and incidence study-2. Soc Psychiatry Psychiatr Epidemiol. 2012;47(2):203–213. doi: 10.1007/s00127-010-0334-8 21197531. 21197531

[53] Chiarotto A, Deyo RA, Terwee CB, et al. Core outcome domains for clinical trials in non-specific low back pain. Eur Spine J. 2015;24(6):1127–1142. Epub 2015 Apr 5. Erratum in: eur Spine J. 2015 Sep;24(9):2097. doi:10.1007/s00586-015-3892-3. 25841358

[54] Edward J, Carreon LY, Williams MV, et al. The importance and impact of patients’ health literacy on low back pain management: a systematic review of literature. Spine J. 2018;18(2):370–376. doi:10.1016/j.spinee.2017.09.005. 28939167

[55] Macfarlane GJ, Pallewatte N, Paudyal P, et al. Evaluation of work-related psychosocial factors and regional musculoskeletal pain: results from a EULAR Task Force. Ann Rheum Dis. 2009;68(6):885–891. doi:10.1136/ard.2008.090829. 18723563

[56] Mather L, Ropponen A, Mittendorfer-Rutz E, et al. Health, work and demographic factors associated with a lower risk of work disability and unemployment in employees with lower back, neck and shoulder pain. BMC Musculoskelet Disord. 2019;20(1):622. 10.1186/s12891-019-2999-9. 31878915 PMC6933729

[57] Teasell RW, Bombardier C. Employment-related factors in chronic pain and chronic pain-related disability. Clin J Pain. 2001;17(4 Suppl):S39–S45. doi:10.1097/00002508-200112001-00010. 11783830

